# Pial arteriovenous fistula with appearance of hemorrhagic tumor: A case report

**DOI:** 10.1097/MD.0000000000022819

**Published:** 2020-10-16

**Authors:** Yuhao Zhao, Yinmeng Qu, Ying Yu, Weikai Yao, Haiyang Xu, Jinlu Yu, Xuan Chen

**Affiliations:** a Department of Neurosurgery; b Department of Neurology; c Department of Pathology, The First Hospital of Jilin University, Changchun, People's Republic of China.

**Keywords:** brain tumor, embolization, pial arteriovenous fistula, resection, thrombosis

## Abstract

**Introduction:**

Pial arteriovenous fistula (PAVF) is a rare intracranial vascular disease, and its presentation with a huge tumor-resembling thrombus is rarer.

**Patient concerns:**

A 38-year-old female patient presented with a sudden left-side motor disorder and loss of consciousness. The patient was otherwise in good health and had no history of hypertension or diabetes. During the physical examination, she appeared lethargic and manifested left limb paralysis with level zero muscle strength and a positive pathological reflex.

**Diagnoses:**

Because imaging failed to rule out a tumor stroke, an intracranial lesion resection was performed immediately. Because the lesion was considered to be a vascular structure, digital subtraction angiography was undertaken before the surgery, and PAVF was diagnosed.

**Interventions:**

Endovascular embolization was conducted, followed by PAVF and hematoma resection.

**Outcomes:**

At the 3-month follow up, her left limb muscle strength was level 4, and she could live on her own (Modified Rankin Scale score = 2).

**Conclusions:**

It is noteworthy that PAVF with a large thrombus may appear as a tumor in the initial diagnosis, and therefore it is necessary to perform an intracranial vascular examination in patients with tumor stroke symptoms.

## 1 Introduction

Pial arteriovenous fistula (PAVF), a rare vascular abnormality's key feature is the direct communication between the intracranial arterial and venous systems without an intervening nidus.^[1,2]^ PAVF is associated with high morbidity and mortality, thus justifying treatment in most cases.^[2]^

It can cause a series of neurological symptoms such as headaches, hemorrhage, seizures, focal neurologic deficits, and raised intracranial pressure.^[3]^ In most cases, hemorrhage can manifest as an intracranial hematoma or subarachnoid hemorrhage.^[3,4]^

In this report, we present a rare case of PAVF with a large thrombus, which caused a mass effect and appeared as a hemorrhagic tumor in the imaging results. A literature review of similar reports was also performed.

## 2 Case presentation

A 38-year-old female patient presented with a sudden left-side motor disorder and loss of consciousness. The patient was otherwise in good health and had no history of hypertension or diabetes. During the physical examination, she appeared lethargic and manifested left limb paralysis with level zero muscle strength and a positive pathological reflex.

**Figure 1:**
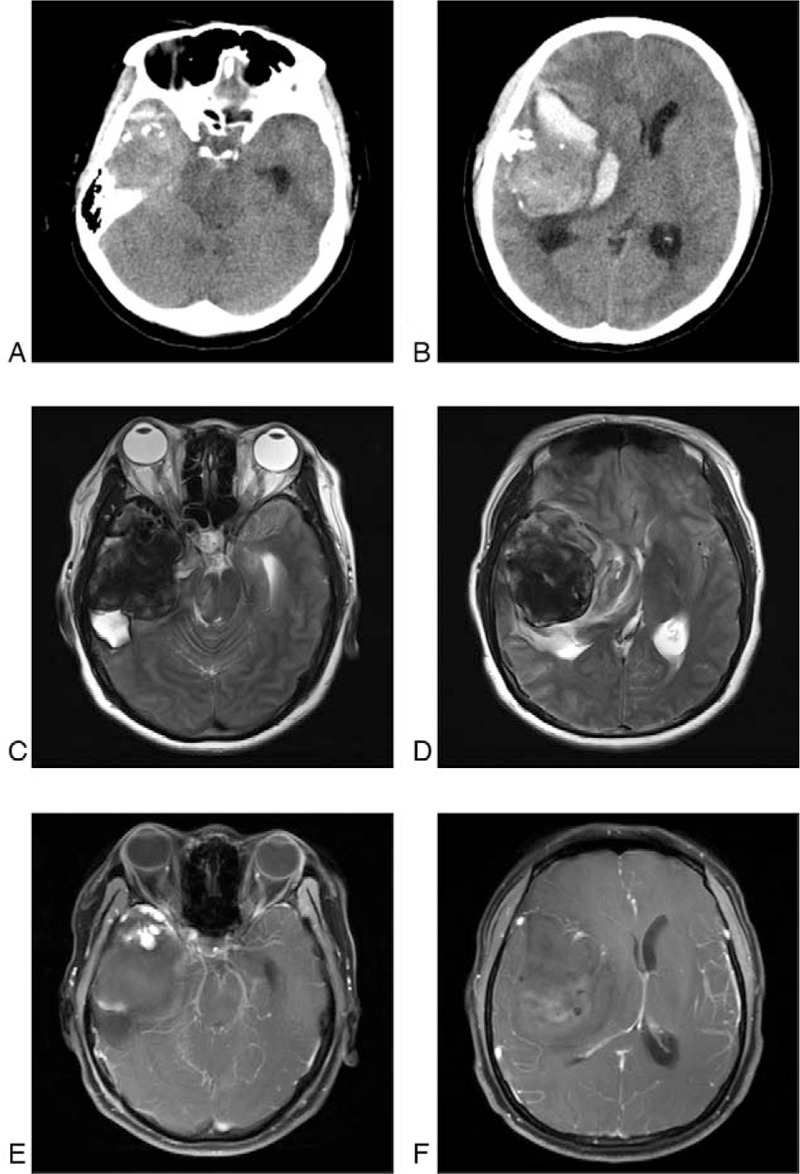
Computed tomography and magnetic resonance imaging. A, B, Right temporal lobe space-occupying lesions with hemorrhage. The ipsilateral ventricle was compressed, and the midline structure was offset to the contralateral side. C, D, right temporal lobe space-occupying lesion with uneven signal surrounded by edema, and vascular flow signal. E, F, the lesions in an enhanced vascular image using magnetic resonance contrast agent enhancement. Inside the lesion, low signals of filling defect were observed.

Head computed tomography findings showed lesions with hemorrhage occupying the right temporal lobe space, compression of the ipsilateral ventricle, and the midline structure offset to the contralateral side (Fig. 1A,B). Further, magnetic resonance imaging examination revealed a right temporal lobe space-occupying lesion with an uneven signal surrounded by an edema and a vascular flow signal. After contrast agent enhancement, lesions were observed in the enhanced vascular image. Inside the lesion, low signals of filling defect were detected suggesting thrombus (Fig. 1C,F).

**Figure 2:**
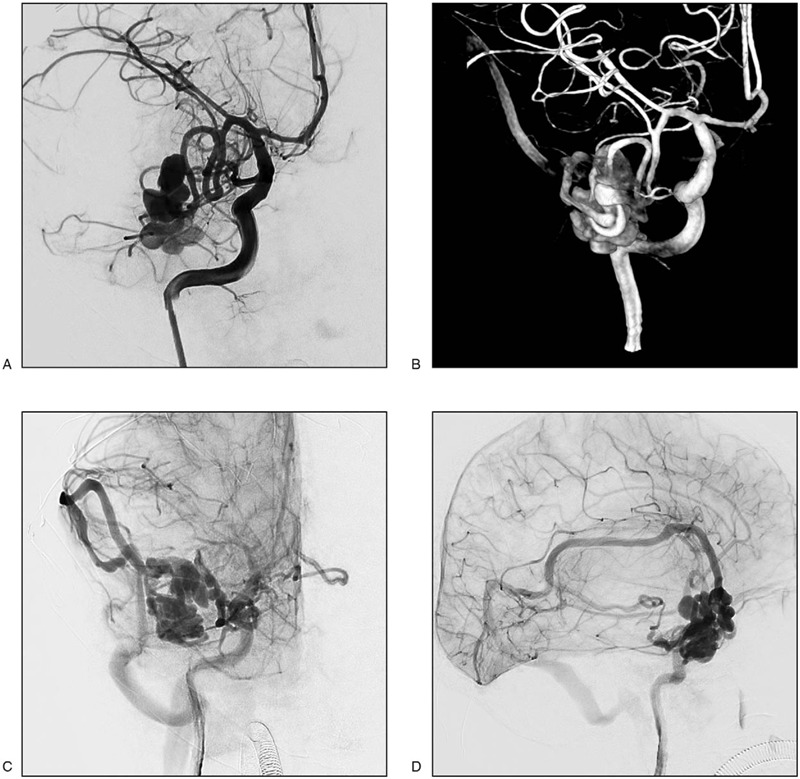
Digital subtraction angiography examination. The pial arteriovenous fistula was fed by the lower branch of right middle cerebral artery. Blood flow through the lateral fissure vein and Labbé vein to the sigmoid sinus. A, Two-dimensional **(**2D) arterial phase image. B, Three-dimensional image. C, D, 2D venous phase.

Intracranial lesion resection was undertaken because the patient was diagnosed with a right temporal lobe tumor with hematoma. The operation was performed under general anesthesia using a right temporal operative approach. A reddish cystic mass was observed on the surface of the brain the dura was opened. It felt tough and had a large number of nourishing arteries on its surface. Because the lesion was considered to have a vascular structure, we immediately closed the skull and transferred the patient to the catheter room for digital subtraction angiography. The angiography showed that the lesion was a PAVF fed by the right middle cerebral artery. Blood flow was through the lateral fissure vein and Labbé vein to the sigmoid sinus (Fig. 2).

**Figure 3:**
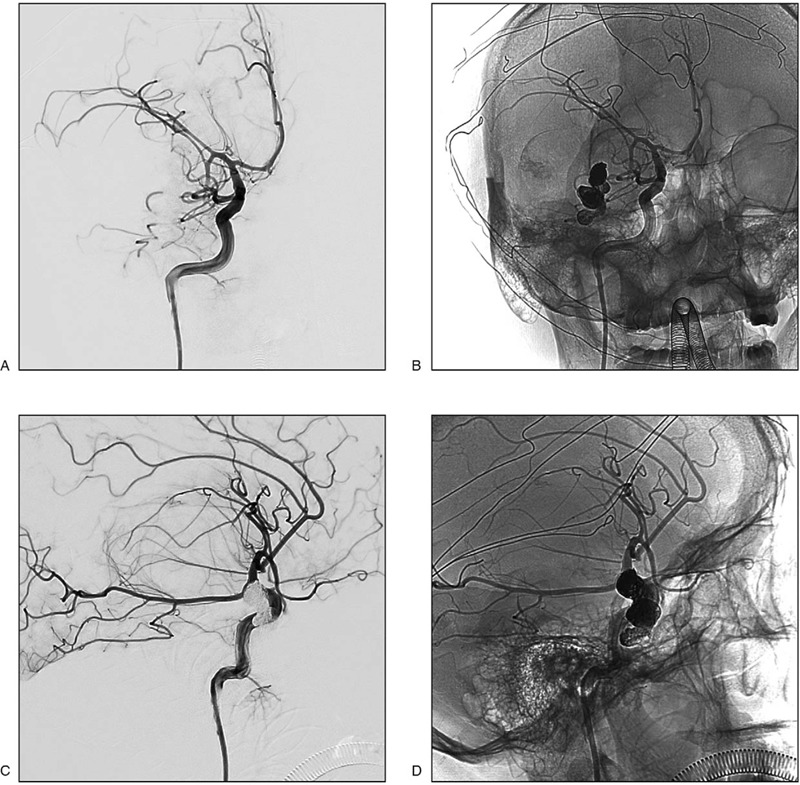
The embolization of pial arteriovenous fistula (PAVF). After embolization of the lesion using coils and Onyx, digital subtraction angiography showed complete disappearance of the PAVF structure. A, Two-dimensional (2D) image showed complete disappearance of the PAVF structure (positive position). B, 2D image showed the shape of Onyx and coils (positive position). C, 2D image showed complete disappearance of the PAVF structure (lateral position). D, 2D image showed the shape of Onyx and coils (lateral position).

**Figure 4:**
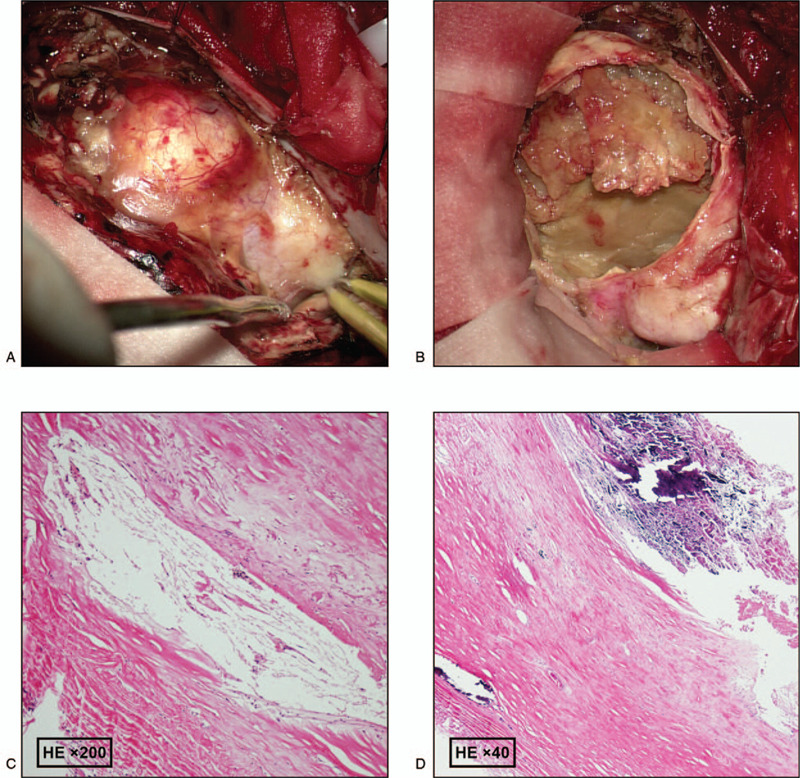
Intraoperative and pathological images. A, a reddish cystic mass on the surface of brain and many nourishing arteries are visible. B, after cutting the saclike structure, a fish-like thrombus can be seen inside. C, D, Pathological results showed attachment of several clots to the intima of the lesion's vessel wall and accumulation of hemosiderin-phagocytosing tissue cells. The medial membrane manifested hyaline and mucoid degeneration, and focal calcification. The extravascular membrane is scattered with chronic inflammatory cells.

The PAVF was completely embolized with endovascular treatment using coils and onyx (Fig. 3). Subsequently, the patient was transported to the operating room for removal of the PAVF and release of the mass effect. During the surgery, it was observed that the PAVF mainly comprised blood clots, and these structures, including the hematoma, were removed (Fig. 4 A,B).

Postoperative pathological results showed attachment of several clots to the intima of the lesion's vessel wall and accumulation of hemosiderin-phagocytosing tissue cells. The medial membrane manifested hyaline and mucoid degeneration, and focal calcification. The extravascular membrane was scattered with chronic inflammatory cells. All these features were consistent with the pathological changes of intravascular thrombosis (Fig. 4C,D).

The patient gradually recovered postoperatively. After 1 week, the patient was discharged, and her left limb muscle strength was level 2. The patient was transferred to another hospital for further rehabilitation training. At the 3-month follow up, her left limb muscle strength was level 4 and she could live on her own (Modified Rankin Scale score = 2).

## 3 Ethics statement

Our institution does not require formal ethical approval for case reports. Written informed consent was obtained from the patient and her parents for publication of this case report.

## 4 Discussion

PAVF is a rare vascular abnormality that accounts for about 1.6% of all intracranial vascular malformations, and is most common in children.^[1]^ The most important anatomical feature is the direct connection between the intracranial arterial system and the venous system of the Willis ring or its secondary branch vessels.^[4,5]^ PAVF can cause headaches, intracranial hemorrhages, seizures, and focal neurological deficits; hemorrhages generally manifest as intracranial hematoma or subarachnoid hemorrhage.^[3,4]^ In this case, the patient had a ruptured PAVF with a large thrombus. It caused a mass effect which made it appear as a hemorrhagic tumor in the imaging results, a phenomenon rarely reported.

Pial AVF can be congenital, iatrogenic, or caused by trauma, and it enables direct communication between arteries and veins.^[2]^ Most pediatric patients are considered congenital, and multiple PAVFs may be associated with hereditary hemorrhagic telangiectasia.^[6]^ This patient was a middle-aged woman without any history of trauma or surgery and had no clinical before the onset of the disease. Congenital cause of formation of PAVF could not be excluded.

The occurrence of thrombus in PAVF is rare, and its cause is still unclear; induction mechanism similar to the thrombosis in giant aneurysms is considered a leading possibility. Slow blood flow and turbulence in the PAVF may lead to endothelial cell dysfunction, resulting in contact between the matrix proteins and blood flow, subsequent activation of the coagulation cascade and eventual thrombus growth. The thrombus can further lead to cell lysis and release of thrombin, thereby contributing to thrombus growth again.^[7,8]^

In addition, the wall of a giant aneurysm contains nourishing vessels; thus, the intracapsular thrombus can continue to grow even after the aneurysm is embolized.^[9,10]^ This is consistent with the abundant distribution of blood vessels observed on the surface of the lesion in our case. Therefore, the existence of nourishing arteries may also be responsible for the continued growth of the thrombus. Furthermore, pathological studies have already demonstrated that inflammatory responses play a key role in thrombosis of giant aneurysms and arteriovenous malformations.^[11–13]^ In this case, inflammatory cell infiltration was also observed in the vessel wall. It is speculated that inflammation may also be the cause of giant thrombus formation in PAVF.

PAVF is characterized by high disability and mortality rates, therefore, surgical intervention should be performed actively, especially in cases with bleeding symptoms.^[2]^ It is not difficult to diagnose PAVF alone. Most patients with intracranial hemorrhage will have a computed tomography angiography or digital subtraction angiography examination to determine the cause of bleeding. However, in this case, there was a large thrombus in the PAVF, which had a mass effect and resembled as a hemorrhagic tumor, thereby causing an easy misdiagnosis.

In most cases, microsurgery or endovascular embolization is a simple and effective way to disconnect the abnormal connections between the PAVF arterial system and the venous system.^[14]^ To reduce the probability of recurrence due to the existence of nourishing vessels, PAVF should be removed during microsurgery, especially in patients with mass effect.^[13]^ PAVF's embolization before microsurgery is more conducive to its removal; therefore, it was not difficult to remove the lesion and thrombus.

It is noteworthy that PAVF with a large thrombus may appear as a tumor in the initial diagnosis, and therefore it is necessary to perform an intracranial vascular examination in patients with appearance of hemorrhagic tumor.

## Author contributions

**Conceptualization:** Yuhao Zhao, Xuan Chen, Jinlu Yu.

**Data curation:** Ying Yu, Weikai Yao, Haiyang Xu.

**Project administration:** Yuhao Zhao.

**Supervision:** Xuan Chen.

**Writing – original draft:** Yuhao Zhao, Yinmeng Qu.

**Writing – review & editing:** Yinmeng Qu, Xuan Chen, Jinlu Yu.
